# P115: Sustained improvement of hand hygiene practice at Tianjin Infectious Disease Hospital, China

**DOI:** 10.1186/2047-2994-2-S1-P115

**Published:** 2013-06-20

**Authors:** B Gao, J-G Dai, J-P Ma, W-Y Zhu, L Liu, W Lu

**Affiliations:** 1 Nosocomial Infection Control Unit, Tianjin Second People's Hospital, Tianjin, China; 2 Graduate School, Tianjin Medical University, Tianjin, China; 3 Tianjin Second People's Hospital, Tianjin, China

## Objectives

To persistently improve HCWs hand hygiene compliance within an infectious disease hospital in China.

## Methods

New technical tools were supplemented and coordinated within a tertiary teaching infectious disease hospital based in 2011 and 2012 on base of the series of interventions conducted during the five preceding years. New tools included training of HCWs as trainer to train hand hygiene to patients and visitors, implementing hand hygiene in Health Education to patients admitted, introducing artistic hand hygiene video play in hospital waiting-hall, and audits being rich efficiency, etc. Hospital-wide one-day prevalence surveys of NI were performed annually by HELICS version 7.0 around the end of October of 2011/12. Hand hygiene opportunity (based on the WHO 5 Moments) data were collected through direct observations.

## Results

During 603.7 hours in 2011 and 657.7 hours in 2012 of observation, 6,690 and 12,442 hand hygiene opportunities were identified respectively. The trends of consumption of alcohol-based hand rub by HCWs of the whole hospital administration units increased from 11.624 L/*1*,*000 patient-days* in January 2011 to 12.578 L/*1*,*000 patient-days* in December 2012 (*p*<0.001). Compliance with hand hygiene increased significantly from 68.52% in 2011 to 83.50% in 2012 (*p*=0.000). The one-day prevalence of NI within the whole hospital decreased from 0.77% (2/260) in 2011 to 0.00% (0/219) in 2012 (*p*=0.010).

## Conclusion

Multi-modal strategies continuously introduced new technical tools were necessary to sustained improvement of hand hygiene compliance with concomitant reduction in nosocomial infections.

## Disclosure of interest

None declared

